# *LRBA* Gene Polymorphisms and Risk of Coal Workers’ Pneumoconiosis: A Case–Control Study from China

**DOI:** 10.3390/ijerph14101138

**Published:** 2017-09-27

**Authors:** Yi Liu, Jingjin Yang, Qiuyun Wu, Ruhui Han, Weiwen Yan, Jiali Yuan, Xiaoming Ji, Yan Li, Wenxi Yao, Chunhui Ni

**Affiliations:** Key Laboratory of Modern Toxicology of Ministry of Education, Department of Occupational Medicine and Environmental Health, School of Public Health, Nanjing Medical University, Nanjing 211166, China; liuyinjmu@163.com (Y.Liu); njmujj@126.com (J.Yang); xjwqy922@163.com (Q.W.); hanruhui007@163.com (R.H.); weiwenyan911016@163.com (W.Yan); yjlgogo@sohu.com (J.Yuan); jixiaominglove@163.com (X.J.); liyan_njmu@163.com (Y.Li); ywx3737@163.com (W.Yao)

**Keywords:** *LRBA*, polymorphisms, coal workers’ pneumoconiosis, genetics

## Abstract

The lipopolysaccharide (LPS)-responsive beige-like anchor protein (LRBA) is a member of the WDL-BEACH-WD (WBW) gene family. Defects in this gene are associated with the disordered autoimmunity in various diseases, including pulmonary fibrosis. In this study, we investigated the association between the functional polymorphisms in *LRBA* and risk of coal workers’ pneumoconiosis (CWP) in a Chinese population. Three potentially functional polymorphisms (rs2290846, rs3749574, and rs1782360) in *LRBA* were genotyped and analyzed in a case–control study, including 703 CWP cases and 705 controls. Genotyping was performed by the ABI 7900HT Real Time PCR system. Our results suggested that genotype rs2290846 AA was significantly associated with decreased risk of CWP (Adjusted OR = 0.61, 95% CI = 0.41–0.92), and the recessive model also supported the protective role of the genotype (Adjusted OR = 0.60, 95% CI = 0.40–0.89). Further, the polymorphism of rs2290846 decreased the CWP risk among cases over 27 years of dust exposure (adjusted OR = 0.51, 95% CI = 0.28–0.94) and non-smokers (adjusted OR = 0.58, 95% CI = 0.34–1.00). A potential role of rs2290846 AA has been proposed by expression quantitative trait loci (eQTL) and The Cancer Genome Atlas (TCGA). The present results suggest that *LRBA* SNPs are associated with CWP susceptibility in a Chinese population. Further studies focused on detailed mechanism or larger cohorts are warranted to validate our findings.

## 1. Introduction

Pneumoconiosis is a group of occupational lung fibrotic diseases which is primarily caused by exposure to inorganic dust particulates which are retained in the lung parenchyma, such as crystalline silica, asbestos, and coal dust [1]. Pneumoconiosis has been the major occupational disease in China in the past 60 years and presently, accounting for more than 70% of new cases annually. As reported in 2014, 89.66% of the total 29,972 reported occupational cases was pneumoconiosis, among which coal workers’ pneumoconiosis (CWP) and silicosis accounted for 51.5% and 42.7%, respectively [2]. CWP is a kind of incurable and progressive disease, characterized by chronic lung inflammation and the formation of fibrotic lesions which result from the inhalation of airborne coal mining dust containing free crystalline silica [3]. Although strong evidence showed that various cytokines and the extracellular matrix (ECM) are involved in the CWP process [4], the pathogenesis and influencing factors of CWP are not entirely clear. 

The pathogenesis of pulmonary fibrosis is quite complex, since numerous molecular pathways and cell types are involved in the process. Activated inflammatory cells, vascular leak, and released profibrotic cytokines create a supportive environment for exaggerated fibroblast and myofibroblast activity [5]. Various cell types participate in this progress, including macrophages, T lymphocytes, vascular endothelial cells, epithelial cells, myofibroblasts, and so on [6]. Altered levels of serum immunoglobulins have been found in CWP since the 1980s [7]. Previous studies proved that abnormalities in T lymphocytes may play an important role in pulmonary fibrosis [8]. T cells could regulate fibrosis by producing cytokines. Treatment with anti-CD4 antibodies reduces the severity of fibrosis, while IL-10 helps limit the silica-induced inflammatory response but amplifies the fibrotic response [9,10]. Additionally, lymphocyte–monocyte interactions have been identified as crucial pathological processes in fibrotic diseases [11].

Autophagy is a highly conserved fundamental mechanism which is mainly involved in the clearance of damaged organelles and proteins to maintain cellular homeostasis [12,13]. Cytosolic substrates are assimilated in autophagosomes and then transferred to endosomes or lysosomes for further digestion, which is induced by lysosomal hydrolases [14]. Studies have proved that autophagy plays an important role in the processes of various diseases, including cancer, diabetes, and fibrosis [15,16,17]. A recent study showed that autophagosomes accumulated in alveolar macrophages of human silicosis [18], and also our previous study proved that autophagy is involved in the development of silica-induced pulmonary fibrosis [19].

Mutations in the “lipopolysaccharide (LPS)-responsive beige-like anchor protein” (*LRBA*) gene cause a syndrome of autoimmunity, lymphoproliferation, and humoral immune deficiency [20]. Meanwhile, LRBA plays a key role in autophage and T-cell deficiency [21,22]. Germline mutations have been found in the *LRBA* gene, which could affect its function by abolishing the expression of LRBA protein [23]. Although it is quite clear that LRBA could regulate autophagy and T-cell function, whether the genetic variations of *LRBA* are associated with the risk of CWP has not yet been explored. In this study, we investigated the potential role of *LRBA* polymorphisms in CWP by a Chinese population to better understand the genetic susceptibility of CWP. 

## 2. Materials and Methods

### 2.1. Study Subjects

This research was conducted in accordance with the Declaration of Helsinki, and the protocol was approved by the Institutional Review Board of Nanjing Medical University (approval code NJMUER201600328). The present case–control study consisted of 703 CWP patients and 705 controls, which were recruited from the coal mines of Xuzhou Mining Business Group Co., Ltd. (Xuzhou, China) between January 2006 and December 2010, as described previously [24]. All subjects are coming from an ethnically homogeneous Chinese population without a genetic relationship who spent their entire working career within the company mentioned above. Subjects were excluded if they had clinical evidence of autoimmune diseases, had received immunosuppressive or immunostimulatory therapy, or were subjected to radiotherapy. High-kilovolt chest X-ray and physical examinations were performed based on the China National Diagnostic Criteria for Pneumoconiosis (GBZ 70-2002) to confirm diagnoses. The pneumoconiosis cases were classified into stage I, stage II, or stage III according to the size, profusion, and distribution range of opacities with agreement by at least two out of three national certified readers. The controls were healthy miners from the same company, matched with the CWP cases for age, dust exposure period, and job types. Through a double-blind method, the questionnaire was done by face-to-face interviewers focused on individual information including age, respiratory symptoms, occupational histories, smoking habits, and others. Blood samples (5 mL) were obtained from all subjects and used for routine lab tests. Written informed consent was obtained from all individuals in this study. Our research protocol was specifically approved by the Institutional Review Board of Nanjing Medical University. The investigations were carried out following the rules of the Declaration of Helsinki of 1975, revised in 2008.

### 2.2. SNP Selection

To select the most likely functional single nucleotide polymorphisms (SNPs) influencing *LRBA* gene, we chose all the SNPs located in the exon as determined in the UCSC Genome Browser (Human GRCh37/hg19). We included the following criteria for SNPs: (i) the SNPs should be located in the exon; (ii) the minor allele frequency (MAF) should be >5% in the Chinese Han Beijing population (CHB); and (iii) the SNPs should be non-synonymous. At last, three SNPs (rs2290846, rs3749574, rs1782360) located in the exon region were included in the study, which were likely to regulate the transcription of *LRBA*.

### 2.3. Genotyping

The genomic DNA was isolated from the peripheral blood samples of the study subjects using the conventional phenol-chloroform method. A 7900HT Real Time PCR system (Applied Biosystems, Foster City, CA, USA) was used to perform genotyping, according to the manufacturer’s protocols. The sequences of primer and probe for each SNP are available on request (BioSteed BioTechnologies Co., Ltd., Nanjing, China). The amplification was performed in a total volume of 5 μL, 50 ng genomic DNA was used for each reaction, and amplification was performed under the following conditions: 50 °C for 2 min and 95 °C for 10 min followed by 45 cycles of 95 °C for 15 s and 60 °C for 1 min. Negative controls were included in each plate to ensure accuracy of the genotyping. Ten percent of the samples were randomly selected for confirmation, and the results were 100% concordant. For quality control, genotyping was performed by two researchers in a blinded fashion. Genotyping was conducted by two researchers independently in a blinded fashion without knowledge of the workers’ personal details or case status.

### 2.4. Bioinformatical Analysis and Gene Expression Levels

Data from the Genotype-Tissue Expression project (GTEx v6, version phs000424.v6.p1, Bethesta, MD, USA) (https://gtexportal.org/home/testyourown) were used to perform expression quantitative trait loci analysis of 278 lung tissues. The expression levels LRBA in 57 pairs of lung adenocarcinoma samples versus their adjacent normal lung tissues were analyzed by The Cancer Genome Atlas (TCGA) database (collaborated between the NCI and the NHGRI, Bethesta, MD, USA) (https://cancergenome.nih.gov/).

### 2.5. Statistical Analysis

The Student’s *t*-test (for continuous variables) or the *χ*^2^ test (for categorical variables) were used to examine the differences of the characteristics for CWP patients and control subjects. For the case–control study, Hardy–Weinberg equilibrium (HWE) was tested by using a goodness-of-fit *χ*^2^-test. The associations between polymorphisms in *LRBA* and risk of CWP were estimated by computing odds ratios (ORs) and their 95% confidence intervals (CIs) from unconditional logistic regression analysis with the adjustment for the possible confounders including age, dust-exposure years, smoking status, and job type. For the stratified analysis, the age and dust-exposure cut-offs used were according to the median age and dust-exposure years of the recruited patients and controls. The statistical power was calculated by the PS software (version 3.1.2, Vanderbilt University, Nashville, TN, USA) (http://biostat.mc.vanderbilt.edu/twiki/bin/view/Main/PowerSampleSize). All statistical tests were two-sided and *p* < 0.05 was considered a significant difference. All data arrangement and statistical analysis were performed with SPSS 13.0 software (Chicago, IL, USA).

## 3. Results

### 3.1. Characteristics of the Study Subjects

The demographic information of cases and controls enrolled in this study is summarized in Table 1. Mean age suggested no significance (*p* = 0.086) between CWP patients (67.3) and control group (66.3). Additionally, the exposure years (*p* = 0.170), smoking status (*p* = 0.088), and job type (*p* = 0.703) of CWP were similar to the controls. However, the pack-years smoked in CWP cases was significantly less than that of controls (*p* < 0.001). The frequency distributions and means of the selected characteristics were matched adequately between cases and controls. Furthermore, the cases of stages I to III were 431 (61.3%), 209 (19.7%), and 63 (9.0%), respectively.

### 3.2. Associations between the LRBA Polymorphisms and Risk of CWP

In Table 2, we listed the primary information and frequencies of *LRBA* polymorphisms. All genotype distributions of control subjects were consistent with those expected from the HWE (*p* ˃ 0.05). The MAF of all three selected SNPs was consistent with that reported in the HapMap database (http://www.hapmap.org). In addition, all three SNPs could lead to different transcription for located in the exon regions. 

Next, a multivariate logistic regression analysis was performed to assess the effect of SNPs on CWP risk (adjusting for age, exposure years, pack-years smoked, and job type). As shown in Table 3, the parameters for the association of SNPs with CWP suggested that only *LRBA* rs2290846 significantly associated with the risk of CWP. No significant difference in two other *LRBA* SNPs (rs3749574 and rs1782360) was observed between CWP patients and healthy controls under any of the genetic models. The analysis under different genetic models revealed that the risk allele decreased the susceptibility to CWP under co-dominant (AA versus GG; OR = 0.64, 95% CI = 0.43–0.96, *p* = 0.030), and the association remained significant after adjusting for age, exposure years, pack-years smoked, and job type. In addition, under the recessive model of *LRBA* rs2290846 (AA versus GG + GA; adjusted OR = 0.60, 95% CI = 0.40–0.89, *p* = 0.011), significant distribution difference was found between CWP patients and controls.

In Table 4, stratification analysis according to exposure years and pack-years smoked was used to assess the associations between *LRBA* rs2290846 and CWP under a recessive model. Data suggested that in the subjects who had more than 27 years of exposure, the individuals of rs2290846 AA genotype had significantly decreased CWP risk compared with GG/GA genotypes (OR = 0.51, 95% CI = 0.28–0.94, *p* = 0.032) (Table 4), while no significant difference was observed in the subjects with less than 27 years of exposure (*p* = 0.237). This AA genotype also decreased CWP risk in the non-smoker population (OR = 0.58, 95% CI = 0.34–1.00, *p* = 0.049). Further analysis between CWP stage and genotypes of rs2290846 also proved that the AA genotype of rs2290846 could function as protective factor—especially in total CWP (OR = 0.60, 95% CI = 0.40–0.89, *p* = 0.011) and stage Ι patients (OR = 0.60, 95% CI = 0.37–0.97, *p* = 0.035). Since the sample size is relatively small, the protective role was not significant in stages II and III groups (Table 5).

### 3.3. Potential Biological Roles of LRBA rs2290846 in CWP

**Figure 1:**
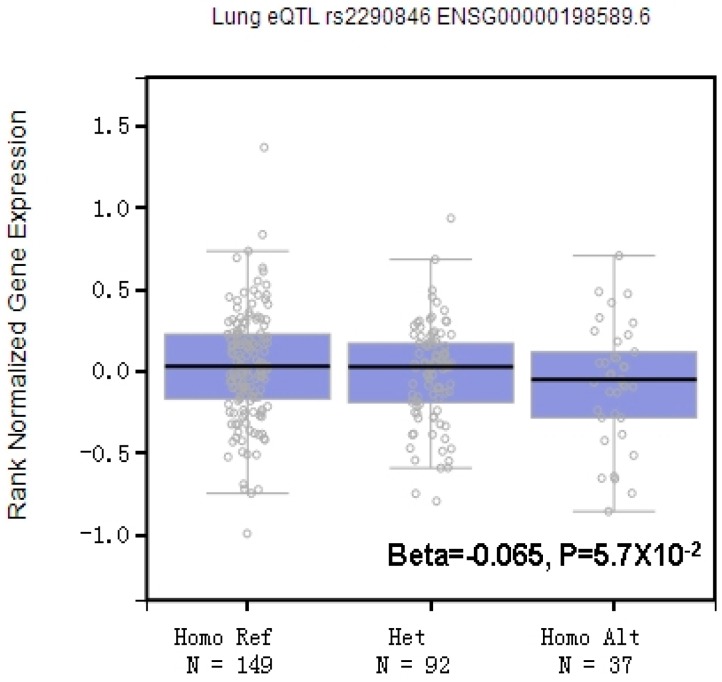
The expression quantitative trait loci (eQTL) analysis of rs2290846 in GTEx.

Since rs2290846 is located in the exon area of *LRBA* and has the potential ability to influence LRBA protein, we investigated the potential biological function of rs2290846 G > A polymorphism. HaploReg v4.1 was used to develop the potential mechanism of the rs2290846 variant on clinical phenotypes and normal variation by searching SNPs with high LD (Supplementary Table S1). To further testify the function of rs2290846, the expression quantitative trait loci (eQTL) was performed by using GTEx Analysis Release V6p (https://gtexportal.org/home/testyourown). The results showed that in the normal lung tissues, the AA genotype of SNP rs2290846 is associated with decreased *LRBA* expression, although the significance is limited (*p* = 0.057) (Figure 1). By using the TCGA database, we analyzed the expression levels of LRBA in 57 lung adenocarcinoma samples and paired adjacent normal lung tissues. The data suggest relatively higher expression of LRBA in lung cancer tissues (*** *p* < 0.001) (Figure S1). These results hinted at a potential protective role of rs2290846 in pulmonary fibrosis.

## 4. Discussion

In the present study, we investigated the potential association between *LRBA* polymorphisms and risk of CWP in a Chinese population. Our results found that *LRBA* rs2290846 G ˃ A—which is located in the exon area of *LRBA*—closely associated with CWP. The phenomenon was significantly observed especially in non-smokers and individuals with longer exposed years. TCGA and eQTL analysis further supported the protective role of the rs2290846 AA genotype by decreasing the level of LRBA protein.

Coal is the major energy source in China [25]. Meanwhile, the occupational disease CWP is usually accompanied with systematic autoimmune diseases. LRBA serves as a key regulator in T-cell function, and mutation in *LRBA* could cause T-cell deficiency and immune dysregulation [21]. However, the germline mutation of *LRBA* has not yet been studied. Recently, the roles of autophagy have been recognized in a variety of diseases. In pulmonary fibrosis, autophagy has been considered as a “cleaner”, and serves to degrade matrix molecules including fibronectin and collagen before they are secreted. Research has proved that autophagy is involved in pulmonary fibrosis and acts as a key point during this process. Meredith et al. considered autophagy as a protective mechanism against pulmonary fibrosis by its effect on myofibroblast differentiation [26], and one of our former studies also supported its protective role [19]. Meanwhile, deficient LRBA has been found to reduce autophagy through lysosome functions [27]. It is quite clear that autophagosomal–lysosomal fusion is one of the most important processes during autophagy. As there are homologs of LRBA and lysosomal functions, mice with mutations in lysosomal proteins have similar phenotypes to those with mutations in the autophagic pathway [27,28]. Taken together, it is reasonable to presume that genetic variations in *LRBA* may affect autophagy in CWP. 

In this study, we revealed *LRBA* gene polymorphisms may have potential influence on the risk of CWP in a Chinese population. Of note, we have identified a significant association with CWP risk at rs2290846 located at 4q31.3, which is the 56th exon region of *LRBA*. This G ˃ A mutation is a missense mutation, converting the serine to leucine, which might influence the expression of LRBA and affect its function. The protective role of rs2290846 AA was further proposed by stratified analysis, but the small sample size of cases in stage II and III restricted our conclusion. By using eQTL, we found that the AA genotype of SNP rs2290846 was associated with a decreased LRBA expression in normal lung. Due to the limited sample size, the *p*-value is not significant, and a larger cohort may better reveal its regulatory role. To further explore the role LRBA played in diseases, we used TCGA lung adenocarcinoma tissues to validate its expression. The results suggested a higher level of LRBA in the pathological tissues, which also supported that rs2290846 may decrease disease occurrence by regulating LRBA expression. Our previous study also suggested that polymorphisms in another autoimmune-related gene (*GIRT*) are strongly associated with CWP [29], which reminds us of the importance of autoimmunity in the process of pulmonary fibrosis. More evidence is needed to verify the role LRBA played during pulmonary fibrosis, and perhaps further findings focused on the tissues of pulmonary fibrosis could shed new light on the therapeutic targets in CWP. To our best knowledge, this is the first research focused on *LRBA* polymorphisms. 

We found that a protective effect of the rs2290846 AA genotype were evident compared with GG/GA. Though the specific underlying mechanisms are not clear, considering LRBA function in autoimmune and autophagy, we still could make a putative educt. Chen et al. suggested that decreased lysosome numbers lead to the increased formation of autophagesomes, exacerbating the apoptosis in alveolar macrophages of silicosis, preventing autophages from benefitting silicosis [18]. It has also been proved that LRBA deficiency could reduce autophagy level in B cells [27]. We proposed that the rs2290846 AA genotype may play an important role in decreased levels of LRBA, which is related to autophagy and reduced risk of CWP. Interestingly, there was no significant difference between smokers and non-smokers related to CWP risk (*p* = 0.088), but a former study showed that smoking was significantly associated with silicosis [30]; this may due to insufficient sample size. In the present study, we also found that the *LRBA* rs2290846 variant played a more obvious protective role in nonsmokers with long dust exposure history and stage Ι CWP. It is possible that cigarette smoke and dust exposure blur the protective role of rs2290846 in CWP risk.

Though we have analyzed the relationship between *LRBA* SNPs and CWP risk, several limitations of our study still exist. First of all, our subjects are coal miners in China, which may cause selection bias. Secondly, more biological background data and functional studies in CWP patients would be quite helpful to explain the correlation between rs2290846 and CWP risk. Furthermore, other environmental exposure or factors (e.g., dietary habits) may be closely associated with *LRBA*, except for cigarette smoke and occupational exposure. Further genome-wide association study with a larger population and relevant molecular mechanism study could help us to improve our understanding about *LRBA* SNPs in CWP. 

## 5. Conclusions

The present study first suggests that *LRBA* rs2290846 polymorphism is associated with CWP susceptibility in a Chinese population. It is also suggested that LRBA might be a potential diagnostic biomarker for CWP. Further studies focused on detailed mechanism or with larger cohorts are warranted to validate our findings.

## Figures and Tables

**Table 1:** Demographic and selected variables among the coal workers’ pneumoconiosis (CWP) cases and controls.

Variables	CWP (*n* = 703)		Controls (*n* = 705)		*p*
	*n*	%	*n*	%	
Age, years (mean ± SD)	67.3 ± 10.8		66.3 ± 9.7		0.086
Exposure years (mean ± SD)	24.9 ± 8.5		25.5 ± 7.1		0.170
Smoking					0.088
No	374	53.2	343	48.7	
Yes	329	46.8	362	51.3	
Pack-years smoked					**<0.001**
0	374	53.2	343	48.7	
0–20	201	28.6	165	23.4	
>20	128	18.2	197	27.9	
Job type					0.703
Tunnel and coal mining	590	83.9	599	85.0	
Transport	40	5.7	42	6.0	
Others	73	10.4	64	9.1	
Stage					
I	431	61.3			
II	209	29.7			
III	63	9.0			

Data with *p* < 0.05 was in bold.

**Table 2:** Primary information of genotyped SNPs in lipopolysaccharide (LPS)-responsive beige-like anchor protein (*LRBA*).

Cluster ID	Region	dbSNP Allele	Function	Protein Residue	MAF		HWE ^a^
					Case	Control	
rs2290846	Exon-56/57	G > A	missense	Ser/Leu	0.256	0.279	0.060
rs3749574	Exon-54/57	C > T	missense	Ala/Thr	0.251	0.275	0.504
rs1782360	Exon-54/57	G > C	missense	Ala/Gly	0.125	0.123	0.091

HWE ^a^ (Hardy–Weinberg equilibrium) *p* value in the control group. MAF: minor allele frequency; SNP: single nucleotide polymorphism.

**Table 3:** Distributions of genotypes of *LRBA* and their associations with CWP risk.

Variables	CWP Cases		Controls		OR (95% CI) ^a^	*p* ^a^	OR (95% CI) ^b^	*p* ^b^
	*n*	%	*n*	%				
**rs2290846**	*n* = 695		*n* = 684					
GG	384	55.3	371	54.2	1.00	-	1.00	-
GA	266	38.3	245	35.8	1.05 (0.84–1.31)	0.677	1.05 (0.84–1.32)	0.680
AA	45	6.5	68	9.9	**0.64 (0.43–0.96)**	**0.030**	**0.61 (0.41–0.92)**	**0.018**
Dominant					0.96 (0.78–1.19)	0.706	0.95 (0.77–1.18)	0.658
Recessive					**0.63 (0.42–0.93)**	**0.020**	**0.60 (0.40–0.89)**	**0.011**
Additive					0.90 (0.76–1.06)	0.196	0.89 (0.75–1.05)	0.152
**rs3749574**	*n* = 698		*n* = 692					
CC	386	55.3	360	52.0	1.00	-	1.00	-
CT	274	39.3	284	41.0	0.90 (0.72–1.12)	0.346	0.90 (0.72–1.13)	0.364
TT	38	5.4	48	6.9	0.74 (0.47–1.16)	0.186	0.70 (0.45–1.10)	0.124
Dominant					0.88 (0.71–1.08)	0.221	0.88 (0.71–1.09)	0.232
Recessive					0.77 (0.50–1.20)	0.249	0.73 (0.47–1.14)	0.162
Additive					0.88 (0.74–1.05)	0.146	0.87 (0.73–1.04)	0.127
**rs1782360**	*n* = 695		*n* = 686					
GG	539	77.6	534	77.8	1.00	-	1.00	-
CG	139	20.0	135	19.7	1.02 (0.78–1.33)	0.883	1.01 (0.77–1.32)	0.943
CC	17	2.4	17	2.5	0.99 (0.50–1.96)	0.979	1.01 (0.51–2.02)	0.971
Dominant					1.02 (0.79–1.31)	0.898	1.01 (0.78–1.30)	0.964
Recessive					0.99 (0.50–1.95)	0.969	1.01 (0.51–2.00)	0.985
Additive					1.01 (0.81–1.26)	0.922	1.01 (0.81–1.25)	0.964

Abbreviations: Dominant, wild homozygote versus heterozygote and mutational homozygote; Recessive, wild homozygote and heterozygote versus mutational homozygote; Additive, wild homozygote versus heterozygote versus mutational homozygote. ^a^ Two-sided *χ*^2^ test. ^b^ Adjusted for age, exposure years, pack-years smoked and job type in logistic regression model. Data with *p* < 0.05 was in bold.

**Table 4:** Stratified analysis between the genotypes of *LRBA* rs2290846 and CWP risk.

Variables	rs2290846			
	Cases ^a^	Controls ^a^	OR (95% CI) ^b^	*p* ^b^
Exposure years				
<27	341/27	355/37	0.73 (0.43–1.23)	0.237
≥27	309/18	261/31	**0.51 (0.28–0.94)**	**0.032**
Pack-years smoked				
0	343/24	302/36	**0.58 (0.34–1.00)**	**0.049**
>0–20	184/16	138/19	0.65 (0.32–1.33)	0.241
>20	123/5	176/13	0.57 (0.20–1.69)	0.313

^a^ Heterozygote+Wild type homozygote/Variant homozygote; ^b^ Adjusted for age, exposure years, pack-years smoked, and job type. Data with *p* < 0.05 was in bold.

**Table 5:** Stratified analysis between the genotypes of *LRBA* rs2290846 and CWP stage.

Variables	Cases/Controls	Genotypes (Cases/Controls)				*p* ^a^	OR (95% CI) ^a^
		AA		GG/GA			
		N	%	N	%		
Total	703/705	45/68	6.4/9.6	658/637	93.6/90.4	0.011	0.60 (0.40–0.89)
Stage							
Ι	431/705	26/68	6.0/9.6	405/637	94.0/90.4	0.035	0.60 (0.37–0.97)
II	209/705	14/68	6.7/9.6	195/637	93.3/90.4	0.137	0.62 (0.34–1.16)
III	63/705	5/68	7.9/9.6	58/637	92.1/90.4	0.381	0.65 (0.24–1.72)

^a^ Adjusted for age, exposure years, pack-years smoked, and job type in logistic regression model.

## Supplementary Materials

The following are available online at www.mdpi.com/1660-4601/14/10/1138/s1. Table S1: High LD SNPs related to rs2290846. Figure S1. The LRBA expression level in lung adenocarcinoma (*n* = 57) and paired adjacent normal lung tissues (*n* = 57). The primary LRBA expression data in lung adenocarcinoma patients were downloaded from the TCGA database (*** *p* < 0.001).
